# Macronutrient in soils and wheat from long-term agroexperiments reflects variations in residue and fertilizer inputs

**DOI:** 10.1038/s41598-020-60164-6

**Published:** 2020-02-24

**Authors:** Santosh Shiwakoti, Valtcho D. Zheljazkov, Hero T. Gollany, Markus Kleber, Baoshan Xing, Tess Astatkie

**Affiliations:** 1 Department of Crop and Soil Sciences, Washington State University, Pullman Washington, United States of America; 2 Department of Crop and Soil Science, Oregon State University, Corvallis Oregon, United States of America; 3 United States Department of Agriculture- Agriculture Research Service, Columbia Plateau Conservation Research Center, Pendleton Oregon, United States of America; 4 Stockbridge School of Agriculture, University of Massachusetts, Amherst Massachusetts, United States of America; 5 Faculty of Agriculture, Dalhousie University, Truro, Nova Scotia Canada

**Keywords:** Plant sciences, Biogeochemistry

## Abstract

Previous studies in the long-term experiments at Pendleton, OR (USA), were focused on organic matter cycling, but the consequences of land management for nutrient status over time have received little attention. Soil and wheat (*Triticum aestivum* L.) tissue samples were analyzed to determine the macronutrient dynamics associated with residue management methods and fertilizer rate under a dryland winter wheat-fallow rotation. The treatments included: no burn residue incorporation with farmyard manure (FYM) or pea vines, no burn or spring burn with application of N fertilizer (0, 45, and 90 kg ha^−1^), and fall burn wheat residue incorporation. The results revealed no differences on the effect of residue burning on macronutrient concentration over time. After receiving the same treatments for 84 years, the concentrations of soil organic C, total N and S, and extractable Mg, K, P in the 0–10 cm depth significantly increased in FYM plots compared to the rest of the plots. The N fertilization rate of 90 kg ha^−1^ reduced the accumulations of P, K, and Ca in grain compared to the 0 and 45 kg N ha^−1^ applications. The results indicate that residue incorporation with FYM can play vital role in reducing the macronutrient decline over time.

## Introduction

Crop yields are no longer increasing at the rate of a couple of decades ago^1^ and, simultaneously, the proportion of arable lands are also constantly shrinking^2^ – a condition that demands increased cultivation under dryland cropping systems. Therefore, examining various soil management practices and their impacts on soil quality, such as macronutrient status, of such regions may contribute to sustainable food production for the next 100 years. Despite the important roles of macronutrients for plant growth, few studies have evaluated their dynamics under dryland cropping systems over extended periods of time.

The drylands of the Pacific Northwest (PNW) receive an average annual precipitation of 150–437 mm^3^, while winter wheat requires 500–580 mm of water to complete its lifecycle^4^, and hence, annual dryland wheat production may not be successful in this region. To overcome this water limitation, farmers in this production region of more than two million hectares have opted to utilize a winter wheat-14 months fallow (WW-F) rotation for over 100 years. This system has so far been proven to be economical and stable^5^. In the WW-F cropping system, wheat is grown for ten months and the land is in fallow for 14 months to conserve winter precipitation for next year’s wheat^6,7^. However, during these 14 months of fallow, the surface is exposed to general degradation, wind erosion, and accelerated C and N losses^6^. Nevertheless, the WW-F cropping system has proven to be the best solution to sustain wheat production in these regions^7^, therefore, management strategies that can maintain or enhance long-term soil productivity are crucial for this region.

Wheat residue burning was a common soil management method during the 1930’s in the WW-F cropping system^6^, and is still being practiced by some farmers in the PNW^8^. The burning of crop residues is favored for better seedling growth, for suppression of plant disease and pest incidences, and to mitigate low soil temperature in the spring^6^. However, repeated burning may decrease SOC, reduce microbial activity, increase CO_2_ emissions, and cause air pollution^9^. The burning of crop residue has decreased soil organic C (SOC) by 20% to 60% within the last 40–50 years^6,10^. In contrast, incorporation of crop residue with N fertilizer and organic amendments has increased SOC storage, N, crop yield and biological activities in this region^6,11^. However, excess N fertilization may be detrimental to soil pH, be economically unjustified, and may result in a net loss of SOC due to enhanced crop residue decomposition^12^. The preceding notion can be debated because some studies have found N fertilization slowed SOC loss compared to no N fertilization^13^. This controversy highlights the uncertainty in the impacts of N fertilization on macronutrients, whereas FYM is ubiquitously known for its positive impacts on macronutrient levels^12^. Nevertheless, it is widely recognized that improved agricultural practices, such as residue and manure incorporation and proper N application, minimize the risk of soil nutrient depletion over time^14^. An understanding of how the above-mentioned crop residue management practices affect soil macronutrient concentrations over time is critical to evaluate the potential success of these practices in the region.

Our study was conducted on one of the several long-term experiments (LTE) maintained by the Columbia Basin Agricultural Research Center (CBARC), near Pendleton, OR. The LTE of our study, hereafter denoted by CR-LTE, was established in 1931 to evaluate the effect of different residue management practices (based on farmers’ practices in the 1930’s) on soil and crop productivity under the WW-F cropping system. Previous studies in CR-LTE demonstrated that N and SOC declined over time^6,8^. Besides N and SOC in the CR-LTE plots, the long-term effects of different residue management methods on other essential plant macronutrients such as P, K, Ca, S, and Mg are unknown. The knowledge of respective effects on soil macronutrients over time will provide insights into the sustainability of these management practices. This knowledge can then be utilized to fine-tune the systems and make them more resilient and sustainable for crop production. However, the effect of agricultural practices on nutrient status takes decades to manifest^6^. Under such conditions, only the analysis of archived soil and crop samples from the LTE can provide the resources to detect subtle changes in soil and crop nutrient status over time caused by the different management practices. We aimed to contribute to the limited knowledge of plant macronutrient dynamics in the soil and wheat grain from drylands of the PNW as affected by residue management. The objectives of this study were (i) to quantify the changes in macronutrients and soil pH brought about by different residue management methods and fertilizer inputs after applying the same treatment for at least 64 years and (ii) to determine the trends, if any, in the macronutrients and soil pH over a 20 year time (1995-2005-2015).

## Results and Discussion

The main and interaction effects for total N, S, and SOC, and extractable P, K, Ca, Mg, and soil pH are presented in the ANOVA table (Supplementary Table S1).The concentrations of studied macronutrients and soil pH did not show evident trends as a function of residue management methods over 20 years (1995-2005-2015), but the macronutrients concentrations and pH differed among the treatments mostly in the top 10 cm soil depth in 2015. Thus, we mostly discussed the macronutrient status and soil pH in the upper 10 cm soil surface in year 2015 i.e. after the application of same treatments for 84 years.

### Soil pH

**Figure 1:**
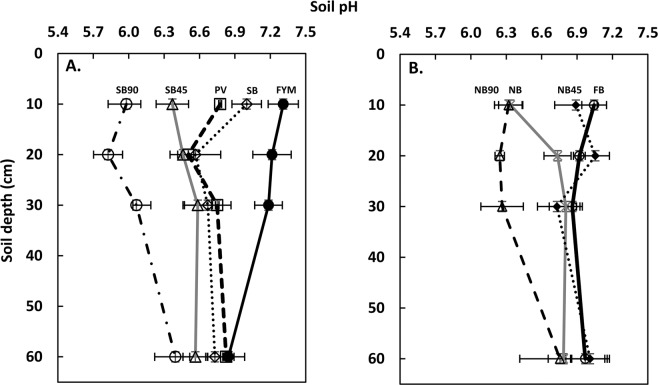
Soil pH depth function as affected by the different agricultural practices of CR-LTE in 2015. (**A**) PV, FYM, SB, SB45, and SB90. (**B**) FB, NB, NB45, and NB90. FB = Fall burn; FYM = Farmyard manure; PV = Pea vine; NB = No burn; SB = Spring burn; and 45 and 90 after NB and SB represents the rates (kg ha^−1^) of inorganic N applications in respective treatments.

After 84 years of WW-F rotation, FYM plots had markedly greater soil pH than the rest of the CR-LTE treatments in the 0–30 cm soil depth (Fig. 1A,B). The addition of FYM replenishes the soil with basic cations and maintains soil pH. Soil pH at top 20 cm soil depth was significantly lower in the NB90 and SB90 than in the FYM, PV, FB, SB, NB45, and SB45 which can be attributed to the greater nitrification from the ammoniacal N fertilizer in NB90 and SB90 than others. Greater acidity was observed at the 10–20 cm soil depth than the other soil depths in the NB90 and SB90 plots, possibly due to the fertilizer placement in that depth (Fig. 1A,B). We did not observe differences in soil pH between residue burn and no burn plots (excluding FYM and PV plots).

### Soil organic carbon (SOC) and total N

**Figure 2:**
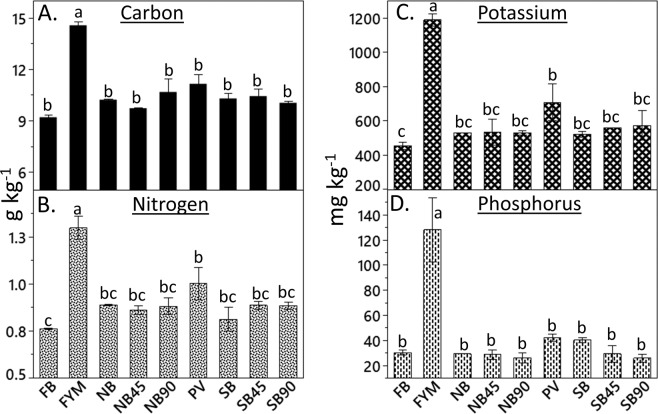
The effect of 84 years of residue management methods on soil nutrients in upper 10 cm soil surface. (**A**) Concentration of soil organic carbon, (**B**) Concentration of total nitrogen, (**C**) Concentration of Mehlich III extractable potassium, and (**D**) Concentration of Mehlich III extractable phosphorus. Bars sharing the same letter are not significantly different at the 0.05 probability level. FB = Fall burn; FYM = Farmyard manure; PV = Pea vine; NB = No burn; SB = Spring burn; and 45 and 90 after NB and SB represents the rates (kg ha^−1^) of inorganic N applications in respective treatments.

Although we did not observe any significant trends for macronutrients over the time, remarkably high concentrations of macronutrients were observed in FYM plots. The manure used in FYM plots supplied an average (10 years average) of 1482, 111, 31, 27 kg ha^−1^ of C, N, P, and S respectively^6^. In addition to this, typical solid livestock manure contains 2.3, 0.26, and 0.51% of K, Ca, and Mg respectively^15^. In 2015, the concentration of SOC in the 0–10 cm depth was significantly higher in FYM (14.6 g kg ^1^) than in the other treatments which were similar to each other (Fig. 2A). Similarly, the concentration of N in the top 10 cm was significantly greater in FYM than in the rest of the plots (Fig. 2B). At 0–10 cm depth, the FYM had a nitrogen concentration of 1.30 g kg^−1^ whereas FB, which had the lowest N concentration, had 0.76 g kg^−1^. The FYM plots received extra amounts of carbon and nitrogen along with the other nutrients which can be attributed to the greater concentrations of SOC and N in the FYM plots than the rest of the plots. This increase in N and SOC concentration can be corroborated by the fact that more organic C input to the soil increases the accumulation of organic N^16^ and reduces its mineralization^17^. Previous research has shown that manure incorporation increases the soil organic matter and the levels of soil macronutrients and their availability^18–21^.

The decrease in the concentrations of SOC and N in these plots was reported previously^8^. Similarly, other researchers reported significant SOC and N declines during the 1990s in all the CR-LTE plots except in the FYM plots^6,22^. Machado *et al*.^23^ reported a significant decline in SOC over the time in 0–60 cm depth profile when compared to the SOC level from 1976 to 2005 in the same experiment. However, our results did not show a decreasing trend in 20 years’ time period for any studied nutrients in the CR-LTE plots. With this contradictory finding in SOC between our study and the previous studies in CR-LTE, we can speculate that the 20 years’ time period may be a short time period to manifest significant changes in the nutrient’s dynamics. In semiarid climate like our research site, ecological processes are very slow and takes more than couple of decades to detect marked change in SOC^6^.

According to Rasmussen *et al*.^22^, the application of inorganic N increases SOC compared to no N application because of its positive effect on the amount of crop residue produced. However, we did not observe significant differences in the SOC and soil N concentrations as a function of inorganic N application although significant grain yield differences in these plots were reported for different N application rates^6^. Similarly, Camara *et al*.^11^ reported significant yield differences in different N application rates in other LTE at CBARC. The results from this study agree with a study by Ghimire *et al*.^8^ who reported that varying N fertilizer application rates did not affect the concentrations of N or SOC. The plausible reason for unresponsive SOC or soil N to inorganic N application could be due to a rapid decomposition of crop residue in N applied plots compared with the plots without N application. The high N content of the crop residue enhanced residue decomposition when incorporated into the soil^6,24^. As a result, crop residue contribution was insufficient to significantly impact SOC and soil N in the SB45, SB90, NB45, NB90, SB, and NB plots^24^. In addition to the rapid decomposition of high N residue, fourteen months fallow period aggravate the depleted SOC pool by depriving the pool with organic matter for prolonged time.

### Mehlich III extractable phosphorus (P) and potassium (K) in soil

The concentration of K was significantly higher in the FYM plots than in the rest of the CR-LTE plots (Supplementary Table S2). Potassium in the 0–10 cm soil depth of the FYM plots increased compared to the other CR-LTE plots in 2015 (Fig. 2C). The FYM and PV plots received more K with the addition of organic matter (OM) than the rest of the plots, but K concentrations were significantly higher only in FYM plots (Supplementary Table S2). The FYM plots had 37% greater K concentration in the top 10 cm soil than the K at the same soil depth in PV. The increased concentration of K with the addition of FYM had been reported in other studies^25,26^. Since manure contains high K, repeated application of manure in the FYM plots could have maintained or increased extractable K compared to the other CR-LTE plots. Another plausible reason for this is that organic manure triggers cation exchange sites to release organic colloids which in turn attracts K from non-exchangeable pools and eventually increases the K availability^27^.

The concentration of P in FYM plots was significantly higher at the 0–10 and 10–20 cm soil depths than in the rest of the CR-LTE plots for all the studied years (Supplementary Table S3). In 2015, phosphorus concentration in the top 10 cm soil depth was 80% higher in FYM plots than in SB90 plots which had the lowest P among the treatments (Fig. 2D). The reason for greater concentration of extractable P in the FYM plots than the rest of the CR-LTE plots is due to the manure application in the FYM plot. Generally, when manure application is based on N requirement by crop, P and K will be over applied^28^. Another reason for increased P in FYM plots could be due to the decreased P adsorption to mineral surfaces and improved microbial population with manure addition, which enhances release of readily available P^29^. Overall, the concentration of extractable P at all studied depths was in the following order: FYM > PV > NB90, with the other treatments being between PV and NB90 (Supplementary Table S3).

### Soil total Sulfur (S) and Mehlich III extractable magnesium (Mg)

**Figure 3:**
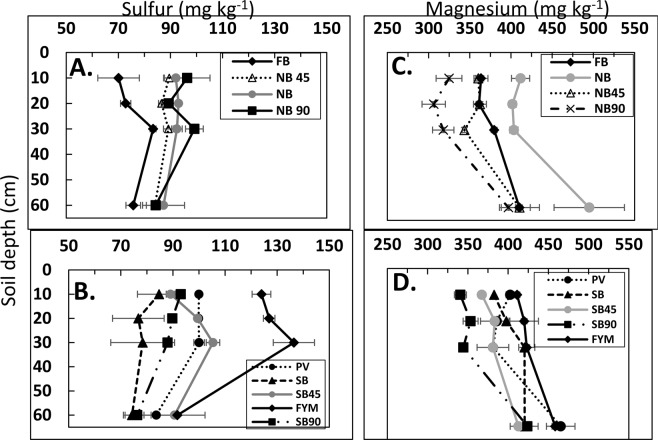
Soil depth function of total sulfur in 2015. (**A**) FB, NB, NB45, and NB90 plots, (**B**) PV, FYM, SB, SB45, and SB90 plots. Soil depth function of Mehlich III extractable magnesium in 2015. (**C**) FB, NB, NB45, and NB90 plots and (**D**) PV, SB, SB45, SB90, and FYM. FB = fall burn; FYM = Farmyard manure; NB, NB45 and NB90 = No burn with N applied at 0 kg ha^−1^, 45 kg ha^−1^, and 90 kg ha^−1^, respectively; PV = pea vine; SB, SB45 and SB90 = spring burn with N applied at 0 kg ha^−1^, 45 kg ha^−1^, and 90 kg ha^−1^, respectively.

Among CR-LTE plots, FYM had higher S concentration than the other plots at all depths except in the 30–60 cm depth in 2015 (Fig. 3A,B). Greater S under FYM treatment is plausible because S is an integral part of OM and FYM received more organic matter than the other plots. All other CR-LTE treatments were comparable in S concentration at 30–60 cm depth (Fig. 3A,B).

In the 0–10 cm soil depth, the concentration of extractable Mg was greater in the FYM and PV plots than in the SB45, SB90, NB45, and NB90 plots (Fig. 3C,D). The higher amount of extractable Mg in FYM and PV plots could be attributed to the addition of OM through manure or pea vines that directly contributes to the soil Mg. The higher concentration of Mg in 30–60 cm soil depth than in the topsoils could be due to the displacement of Mg by K. Due to the competition between these ions for exchange sites, with K having the larger molecular size than Mg, potassium ion can easily displace Mg^30^. The concentration of K was greater in topsoils than in subsoils in this study and so, Mg could have been moved to lower soil depth (Supplementary Table S2).

We did not observe differences in soil macronutrients between residue burn and no burn plots (excluding FYM and PV plots) (Supplementary Tables S2–S5). Perhaps the more favorable seedbed temperature and reduced insect/disease incidence in burned plots during germination may have offset the loss of nutrients to the atmosphere caused by burning. Increased biomass yield can be expected from the favorable seed bed and reduced insect/pest incidence. The biomass eventually decomposes and releases several nutrients. However, this benefit of burning could be annulled when residue is burned which increases nutrient loss to the atmosphere. Nevertheless, burning of residue, especially fall burning, is not recommended in the inland PNW as farming in most areas is performed on 8 to 30% slopes (some slopes as steep as 45%) and soils on such slopes can be vulnerable to erosion in the absence of cover for a prolonged period. Burning of residue could also result in erosion-induced nutrient loss over time.

### Total concentration of nutrients in wheat grain and straw

There were main and interaction effects of year and treatments on the nutrient accumulation in wheat grain and straw (Supplementary Table S6). Significant treatment effects were observed for total concentrations of C, P, K, Ca, and Mg in wheat grain. Total concentration of C in wheat straw was affected by the treatment. Only the significant effects are discussed below.

#### Wheat tissue nitrogen (N) and sulfur (S)

**Figure 4:**
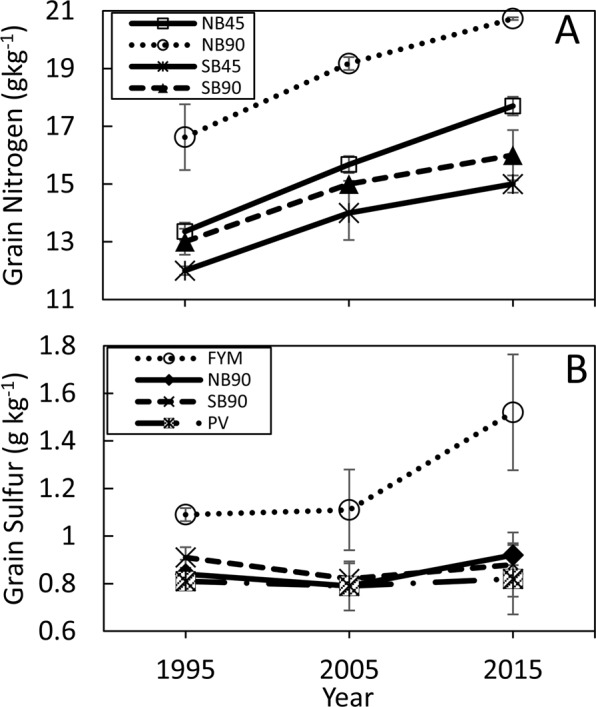
Effect of nitrogen (N) rates on grain N over time (**A**) and effect of organic amendments and N on grain S over time (**B**). FB = fall burn; FYM = Farmyard manure; NB, NB45 and NB90 = No burn with N applied at 0 kg ha^−1^, 45 kg ha^−1^, and 90 kg ha^−1^, respectively; PV = pea vine; SB, SB45 and SB90 = spring burn with N applied at 0 kg ha^−1^, 45 kg ha^−1^, and 90 kg ha^−1^, respectively.

Nitrogen and sulfur are two important nutrients for wheat grain and straw because the concentration of N and S determines grain and straw quality. Higher grain N was found in the NB90 treatment compared with plots without inorganic N application (FB, SB, and NB), while N in grain in the NB90 plots was comparable to that in the FYM plots (Supplementary Table S7). The NB90, SB45 and SB90 plots had similar grain N concentrations in 2015 (Supplementary Table S7). In this study, grain N linearly increased with time (Fig. 4A), which could possibly be due to the less rainfall during the 2015 growing season compared with the rainfall in 2005, which was also lower than the rainfall in 1995 (Supplementary Fig. S1). Water stress increases N in grain^31^.

Grain S of 1.2 mg S g^−1^ is considered as the minimum threshold limit for S deficiency in wheat^32^. The concentrations of S in grain were noticeably higher in FYM plots than the rest of the CR-LTE treatments, and increased from 1995 to 2015 (Fig. 4B and Supplementary Table S7). Only FYM plots had S above the measurement threshold level. Wheat grain has a higher pearling index and decreased dough elasticity when grain S is below the threshold level, which is not a desired trait for marketable grain^33^.

#### Wheat tissue carbon (C) and phosphorus (P)

Carbon concentration in wheat grain was affected by treatments and was greater in the SB45, SB90, NB45, NB90 than in FB and SB (Table 1). The accumulation of N in grain has positive correlation with C in the grain^34^, and SB45, SB90, NB45, NB90 plots had greater N than in FB and SB plots. This could be the reason for greater concentrations of C in the N applied treatments than in the plots without N application. Regarding C in the straw, FYM plots had the highest concentration of C among the CR-LTE (Table 1) suggesting the more pronounced effect of organic N (manure) on C in straw than from inorganic N.

**Table 1:** Mean concentration of significantly affected nutrients in the wheat grain after 84 years in the crop residue long-term experiment (CR-LTE), Pendleton, OR.

	<--------------------------------Grain (g kg^−1^) ----------------------------->					Straw (g kg^−1^)
Treatment	Carbon	Phosphorus	Potassium	Calcium‘	Magnesium	Carbon
NB^a^	442 ab	2.78 b	3.84 ab	0.34 a	1.07 ab	425 ab
NB45	442 a	2.51 d	3.41 c	0.29 bc	1.03 bc	426 ab
NB90	442 a	2.54 d	3.25 c	0.29 bc	1.03 bc	424 b
FB	440 c	2.75 bc	3.85 ab	0.34 ab	1.08 ab	427 ab
SB	441 bc	2.74 bc	3.77 b	0.33 a	1.05 bc	424 b
SB45	443 a	2.42 d	3.33 c	0.28 c	1.00 c	424 b
SB90	442 a	2.59 cd	3.21 c	0.29 bc	1.05 bc	425 ab
PV	441 ab	2.73 bc	3.67 b	0.31 b	1.05 bc	426 ab
FYM	442 ab	3.21 a	4.00 a	0.35 a	1.12 a	431 a

Means sharing the same letter within the columns are not significantly different at 0.05 probability level.

^a^NB = No burn, SB = spring burn, FB = fall burn, FYM = farmyard manure, and PV = pea vine. 0, 45, 90 accompanied by NB, SB, and FB represents rates of N applied at 0 kg ha^−1^, 45 kg ha^−1^, and 90 kg ha^−1^, respectively.

The concentration of P in grain under the FYM (3.21 g kg^−1^) treatment was significantly higher than that under the rest of the treatments (Table 1). Nitrogen application decreased grain P in both the spring burn and no burn treatments (Table 1), which could be due to the low soil pH in the inorganic N applied plots. Wang *et al*.^27^ reported higher P in manure treated soil and subsequently higher P uptake by wheat in FYM treatment. The results from this study agree with the preceding report, as FYM had highest soil P and the highest grain P.

#### Wheat tissue total cations (K, Ca, and Mg)

Organic and inorganic amendments affected the concentrations of K, Ca, and Mg in grain (Table 1). The FYM plots had higher concentrations of K, Ca, and Mg than in the inorganic N applied plots (Table 1). The results indicate that inorganic N affected the absorption of cations, possibly by the competition of NH_4_ + (UAN) with K, Ca, and Mg (cations) on the exchange sites and also due to the decreased soil pH. Consequently, low concentrations of K, Ca, and Mg were observed under inorganic N applied plots. The decreased availability of cations in the soil solution were reflected in low cation accumulations in the grain of inorganic N applied plots. Another plausible reason for increased cations in the FYM plots compared to non-organic amended plots is due to the presence of cations in FYM. Applying FYM increases cation availability ion the soils over time. There were no differences in K, Ca, and Mg in grain between residue burn plots and no burn plots (Table 1).

The following inferences can be made from the results of this study:

- Inorganic N application does neither increase (i) available soil nutrients nor (ii) tissue concentrations of macronutrients over time compared to FYM, and cannot replace FYM application. However, PV application can be replaced by inorganic N application and vice-versa.
- Excluding FYM and PV plots, both (i) spring burning of plant residues or (ii) no burn treatments have similar impacts on soil and tissue macronutrients over time under dryland WW-F rotation in the PNW.
- Desirable lower protein content of soft white winter wheat can be obtained by spring burning of residues, but not through “no burn” of residues. The NB90 plots had 12% (2.07% N × 5.8) protein in grain, whereas the SB45 and SB90 (9.4% protein) plots maintained the optimum protein content for soft white winter wheat, which is 9–10%.
- Soil acidification increased over time by the application of inorganic N, whether the N is applied to burned or unburned residue plots.

## Conclusions

This study determined the long-term impacts of the inorganic N application, manure, and pea vines, in residue incorporated (either burned or unburned) long-term plots. The results indicated that the incorporation of pea vines does not offer significant benefits for reducing macronutrient decline compared to the application of inorganic N. However, manure application showed superior performance than rest of the treatments in the dryland WW-F cropping system and is necessary to curb the macronutrient decline over time. While manure can substantially improve the soil health, including manure alone to meet the crop’s nutrient demand can be challenging from the economic and environment perspectives. In addition to the application of inorganic N, soil must be replenished periodically with other macronutrients to maintain a healthy soil ecosystem where FYM application can play a vital role. Despite having similar effects on soil macronutrient from both the residue burn and no burn treatments, avoiding residue burning may be a preferable option because of the impact of residue burning on air quality and wind erosion. Nonetheless, residue burning can be a better management practice to manage residue, disease, weeds, and to increase soil temperature under some field conditions compared with no burning. Future studies on physical properties and microbial activities in soils of CR-LTE plots may be needed to offer robust guidance in formulating farming strategies and to quantify other aspects of residue burning such as effects on overall soil health.

## Materials and Methods

### Site description

The experiment site is at the Columbia Basin Agricultural Center (CBARC), 15 km northeast of Pendleton, OR (45°42′N, 118°36′W, 438 m asl). The CBARC is one of the 14 field experiment stations of Oregon State University. This experiment was initiated in 1931 and is one of very few LTE remaining in the Western US. The site has dry summers and wet winters with average annual precipitation of 437 mm (90% of precipitation occurs between November and June)^3^, and the 81-yr (1932–2012) average annual maximum and minimum temperatures are 17.4 °C and 3.06 °C, respectively^35^. According to the Soil Survey Staff, the soil at the experimental site is Walla Walla silt loam (coarse-silty, mixed, superactive, mesic Typic Haploxeroll) derived from loess overlying basalt and is well drained. Its main properties are 18% clay, 70% silt (upper 20 cm), pH: 6.1–7.0, and cation exchange capacity: 18 cmol_c_ kg^−1^.

### Experimental design

The experiment was an ordered block of two identical blocks of nine treatments with two replications, each series representing wheat or fallow phase of the WW–F system. Although this experiment was established in 1931 without randomizing the treatments within each block, extensive investigation of plot by plot soil samples and yield data before the experiment started, and later on, revealed no biases^6^. The size of individual plots is 11.6 m by 40.2 m. The two series were offset by 1 year with the sole purpose of collecting wheat yield and biomass data each year. The wheat cultivars grown since 1992 were: Malcolm from 1992–1995, Stephens from 1996–2005, and ORCF102 from 2006–2015.

### Treatments and field management

The CR-LTE treatments include: fall burning of wheat residue without N addition (FB); spring burning of wheat residue with the addition of N at the rate of 0 kg ha^−1^ (SB), 45 kg ha^−1^ (SB45), and 90 kg ha^−1^ (SB90); no burning of wheat residue with the addition of N at the rate of 0 kg ha^−1^ (NB), 45 kg ha^−1^ (NB45) and 90 kg ha^−1^ (NB90); and no burning of wheat residue with farm yard manure (FYM), or with pea vine (PV) incorporation.

The FYM at a rate of 11.2 Mg ha^–1^ yr^–1^ (Dry matter 47.5%, 0.85 Mg C ha^–1^, and 70 kg N ha^–1^ yr^−1^), and pea vine (PV), at a rate of 1.12 Mg ha^–1^ yr^–1^ (Dry matter 87.8%, 0.41 Mg C ha^–1^ yr^–1^, and 18.5 kg N ha^–1^ yr^–1^), were applied just prior to plowing in the spring of the fallow year. Nitrogen was applied as urea-ammonium nitrate solution (UAN 32%, Poole Chemical, Texline, Texas, US) in October, using shank applicator, one week before seeding wheat in SB and NB treatments. The undisturbed wheat stubble was burned after harvests in September for fall burn treatment (FB0) and in late March-early April for spring burn treatment (SB0, SB45, and SB90). The process of burning is rapid with temperatures reaching 300 °C in the canopy for 3 minutes^6^. The soil is left undisturbed between burning and plowing for 195 or 5 days for fall or spring burn, respectively^6^.

After burning and organic amendment application, the entire experiment field is moldboard plowed 20 cm deep and smoothed with a field cultivator or a tine harrow. Before 2002, wheat was sown at 90 kg ha^−1^ and thereafter, the rate of 92 kg ha^−1^ was used. Between April and October, the field is tilled three to four times with a rod weeder to control weeds and maintain seed-zone moisture.

### Soil sampling and laboratory analysis

Soil sampling and laboratory analysis process were similar to that of previous research of similar kind conducted in CBARC^36–41^. Soil cores collected at the 0–10 cm, 10–20 cm, 20–30 cm, and 30–60 cm depths from 1995, 2005, and 2015 were used in this study. We used archived soil samples of 1995 and 2005 while soil samples of 2015 were collected in the summer of 2015. The soil cores (Internal diameter: 3.6 cm) from two locations (north and south central) within each subplot were collected after wheat harvest using a truck-mounted Giddings Hydraulic Probe (Giddings Machine Company, Inc., Windsor, CO). Soil samples from the two locations within a subplot were then composited. Soil pH values (1:2 soil to 0.01 M CaCl_2_ solution) were measured after a 30 min equilibrium time. The CR-LTE had slightly acidic soil to below 60 cm depth and thus, we assumed the total C determined in this study to be SOC. Earlier studies on this plot have confirmed total C in these plots are SOC^6,8^. We did not measure the soil bulk density, however, previous study on the same LTE had reported similar bulk density among the treatments for individual soil depths (0–10, 10–20, 20–30, and 30–60 cm)^8^.

Visible plant material and debris were removed from the soil samples by sieving. Soil samples were oven dried at 60 °C for 72 hours, and roller milled for 4 hours. Wheat grain and straw samples were collected from the center of each plot and were finely ground. A combustion analyzer (Thermo Finnigan FlashEA 1112 Elemental Analyzer, Milan, Italy) for the 1995 and 2005 samples and a Vario Micro Cube combustion analyzer (Elementar Analysensysteme GmbH, Hanau, Germany) for the 2015 samples were used to determine total C, N, and S in soil and plant tissue. Available concentrations of P, K, Ca, and Mg in soil and the total concentration of these nutrients in plants were determined by inductively coupled plasma-optical emissions spectroscopy (ICP-OES Model #2100 DV, Waltham, Massachusetts, US) following a Mehlich III extraction^42^ of soil samples and dry ashing of plant samples^43^.

### Statistical analysis

The concentrations of total N, S, and C and extractable P, K, Ca, and Mg in soil were analyzed by Repeated Measures Analysis (RMA) of a split-plot design. The year (1995, 2005, 2015) was the whole plot factor, treatment (Nine levels: NB, SB45, SB90, NB45, NB90, FB, SB, FYM, and PV) was the subplot factor; and the response variables were measured repeatedly in space at 4 soil depths (0–10 cm, 10–20 cm, 20–30 cm, and 30–60 cm). In RMA, since the assumption of independence is likely to be violated, the Akaike Information Criterion was used to determine the most appropriate co-variance structure and was incorporated in the model using the Mixed Procedure of SAS^44^. Letter groupings were generated using a 5% level of significance for the main effects and using a 1% level of significance for interaction effects to protect Type I experimentwise error rate from over inflation.

Tissue total concentrations of P, K, Ca, and Mg in grain and straw were analyzed as a split-plot design with two blocks where year (1995, 2005, and 2015) was a whole-plot factor and the 9 treatments were a sub-plot factor. We used the Mixed Procedure of SAS^44^ to analyze the data. For significant (p-value <0.05) effects, multiple means comparisons were completed by comparing the least squares means of the corresponding treatment combinations.

The minor differences in pH represent large differences since it is in a logarithmic scale. The pH data were converted to H + concentration (μmol L^−1^) before analyses to unmask the differences of the treatments. The ANOVA table of pH is based on analysis of H + concentration. For multiple comparisons, original values of pH scale were used.

## Supplementary information

Supplementary information.
