# Supplementary material for: Macronutrient in soils and wheat from long-term agroexperiments reflects variations in residue and fertilizer inputs
Source: Sci Rep. 2020 Feb 24;10:3263. doi: 10.1038/s41598-020-60164-6 (PMC7039891; doi:10.1038/s41598-020-60164-6)
Supplement: Supplementary file 1 — Supplementary information. [file 41598_2020_60164_MOESM1_ESM.pdf]

Macronutrients in soils and wheat from long-term agroexperiments reflect variations in residue and fertilizer inputs

Santosh Shiwakoti[1,2], Valtcho D. Zheljazkov[2*], Hero T. Gollany[3], Markus Kleber[2] & Baoshan Xing[4], Tess Astatkie[5]

Supplementary Table S1. ANOVA table of the main and interaction effects of year, treatment and depth on the concentration of soil total N, soil C, soil total S, extractable P, K, Ca, Mg, and soil pH in the crop residue long term experiment.

**Source of variation**	**N**	**C**	**S**	**P**	**K**	**Ca**	**Mg**	**pH**
Year (Y)	0.14	0.05	0.13	0.84	0.07	0.18	0.08	0.09
Treatment (T)	<0.01	<0.01	<0.01	<0.01	<0.01	0.14	<0.01	<0.01
Y × T	0.94	0.97	0.67	<0.01	0.25	0.07	0.76	0.93
Depth (D)	<0.01	<0.01	<0.01	<0.01	<0.01	**<0.01**	<0.01	<0.01
Y × D	<0.01	<0.01	0.24	0.09	**<0.01**	0.06	**<0.01**	**<0.01**
T × D	<0.01	<0.01	**<0.01**	<0.01	**<0.01**	0.19	**<0.01**	**<0.01**
Y × T × D	**<0.01**	**<0.01**	0.25	**0.04**	0.30	0.79	0.99	0.46

Note: Significant effects (p-value < 0.05) that require multiple means comparison are shown in bold.

Supplementary Table S2. Mean concentration of extractable potassium (mg K kg^{-1}) obtained from the combinations of treatment and depth after 84 years in the crop residue long-term experiment (CR-LTE), Pendleton, OR.

Depth (cm)	Treatment								
	FB[1]	FYM	NB	NB45	NB90	PV	SB	SB45	SB90
0-10	C[2] 472 a[3]	A 1096 a	BC 545 a	BC 550 a	BC 542 a	B 688 a	BC 550 a	BC 557 a	BC 585 a
10-20	C 416 a	A 1129 a	B 519 ab	BC 545 a	BC 557 a	B 659 a	BC 564 a	BC 561 a	B 590 a
20-30	C 480 a	A 1003 b	C 501 ab	BC 511 a	BC 543 a	B 659 a	BC 533 a	BC 527 a	B 553 a
30-60	B 409 a	A 699 c	B 442 b	B 411 b	B 411 b	B 543 b	B 433 b	B 427 b	B 408 b

[1] FB = fall burn; FYM =Farmyard manure; NB, NB45 and NB90 = No burn with N applied at 0 kg ha^{-1}, 45 kg ha^{-1}, and 90 kg ha^{-1}, respectively; PV = pea vine; SB, SB45 and SB90 = spring burn with N applied at 0 kg ha^{-1}, 45 kg ha^{-1}, and 90 kg ha^{-1}, respectively.

[2] Upper case letters are comparison of the nine treatments within each depth, and [3] lower-case letters are comparison of the four depths within each of the nine treatments. Means sharing the same letter are not significantly different at 5% level of significance.

Supplementary Table S3. Mean concentration of soil extractable phosphorous (mg P kg^{-1}) obtained from the 108 combinations of treatment, year and depth after 84 years in the crop residue long-term experiment (CR-LTE), Pendleton, OR.

Depth	Treatment								
(cm)	FB[1]	FYM	NB	NB45	NB90	PV	SB	SB45	SB90
**1995**									
0-10	B[2] 35.7 a[3]	A 83.6 a	B 28.4 a	B 29.4 a	B 28.1 a	B 41.4 a	B 43.9 a	B 24.3 a	B 29.1 a
10-20	B 38.4 a	A 94.4 a	B 30.9 a	B 29.2 a	B 28.6 a	B 44.0 a	B 46.8 a	B 26.9 a	B 29.2 a
20-30	CD 35.6 a	A 79.1 a	BCD 42.9 a	D 33.4 a	CD 34.9 a	B 57.8 a	BC 54.5 a	D 34.3 a	CD 34.6 a
30-60	AB 35.2 a	A 54.1 a	AB 39.6 a	AB 38.3 a	B 24.3 a	A 54.9 a	AB 37.7 a	B 22.3 a	B 31.0 a
**2005**									
0-10	B 38.4 a	A 72.5 a	B 38.3 a	B 33.3 a	B 31.3 a	B 34.5 a	B 36.8 a	B 35.5 a	B 35.6 a
10-20	B 38.3 a	A 75.6 a	B 41.0 a	B 35.2 a	B 34.3 a	B 36.0 a	B 37.3 a	B 38.3 a	B 36.2 a
20-30	BC 36.0 a	A 60.3 ab	BC 32.5 a	BC 34.9 a	C 26.9 a	BC 34.8 a	BC 32.6 a	AB 48.2 a	AB 40.9 a
30-60	B 29.7 a	A 51.0 b	AB 31.3 a	AB 33.4 a	B 23.8 a	B 30.6 a	AB 33.0 a	AB 40.3 ab	AB 39.4 a
**2015**									
0-10	B 30.3 a	A 128.2 a	B 29.4 a	B 28.8 a	B 26.0 a	B 41.9 a	B 40.3 a	B 29.3 a	B 25.8 a
10-20	B 30.6 a	A 147.1 a	B 30.4 a	B 28.4 a	B 24.0 a	B 34.9 a	B 36.5 a	B 23.0 a	B 22.0 a
20-30	B 30.7 a	A 141.8 a	B 31.5 a	B 24.5 a	B 25.6 a	B 35.3 a	B 39.7 a	B 23.1 a	B 26.8 a
30-60	BC 25.7 a	A 61.9 b	BC 35.7 a	BC 27.6 a	C 17.0 a	B 39.9 a	BC 36.5 a	BC 25.0 a	BC 25.2 a

[1] FB = fall burn; FYM =Farmyard manure; NB, NB45 and NB90 = No burn with N applied at 0 kg ha^{-1}, 45 kg ha^{-1},and 90 kg ha^{-1}, respectively; PV = pea vine; SB, SB45 and SB90 = spring burn with N applied at 0 kg ha^{-1}, 45 kg ha^{-1}, and 90 kg ha^{-1}, respectively.

[2] Upper case letters are comparison of the 10 treatments within each year and depth, and [3] lower-case letters are comparison of the four depths within each of the nine treatments. Means sharing the same letter are not significantly different at 5% level of significance

Supplementary Table S4. Mean concentration of soil organic carbon (g C kg^{-1}) obtained from the 108 combinations of treatment, year and depth after 84 years in the crop residue long-term experiment (CR-LTE), Pendleton, OR.

Depth	Treatment								
(cm)	FB[1]	FYM	NB	NB45	NB90	PV	SB	SB45	SB90
**1995**									
0-10	C[2] 10.15 a[3]	A 16.81 a	BC 11.23 a	BC 11.45 a	BC 12.09 a	B 13.09 a	BC 11.26 a	BC 12.31 a	BC 11.95 a
10-20	B 10.40 a	A 15.42 a	B 11.50 a	B 11.25 a	B 12.19 a	B 12.34 a	B 11.51 a	B 11.54 ab	B 11.59 a
20-30	A 8.99 a	A 9.10 b	A 9.76 a	A 8.71 b	A 9.02 b	A 9.45 b	A 9.15 bc	A 9.46 b	A 9.31 b
30-60	A 6.68 b	A 6.81 c	A 6.44 b	A 6.96 b	A 5.41 c	A 6.71 c	A 6.99 c	A 6.52 c	A 6.34 c
**2005**									
0-10	C 9.40 a	A 14.18 a	BC 11.08 a	BC 10.87 a	BC 11.27 a	AB 11.95 a	BC 10.80 a	BC 10.93 a	BC 11.33 a
10-20	B 8.92 a	A 12.11 a	B 9.36 ab	B 9.67 a	AB 10.06 a	AB 10.83 a	B 9.55 a	AB 9.86 a	B 9.45 ab
20-30	A 7.79 ab	A 7.79 b	A 7.47 bc	A 6.69 b	A 9.77 b	A 8.21 b	A 7.27 b	A 6.92 b	A 7.28 bc
30-60	A 5.99 b	A 6.27 b	A 6.01 c	A 5.87 b	A 7.82 b	A 6.79 b	A 6.06 b	A 5.87 b	A 5.96 c
**2015**									
0-10	B 9.19 a	A 14.56 a	B 10.22 a	B 9.72 a	B 10.67 a	B 11.15 a	B 10.29 a	B 10.43 a	B 10.04 a
10-20	B 9.52 a	A 13.16 a	B 10.01 a	B 10.60 a	B 10.43 a	B 11.64 a	B 10.44 a	B 10.26 a	B 9.75 a
20-30	C 8.71 a	A 13.98 a	C 9.04 a	BC 10.53 a	BC 10.73a	AB 11.83 a	BC 10.33 a	BC 10.35 a	BC 9.87 a
30-60	A 6.14 b	A 6.96 b	A 6.43 b	A 7.28 b	A 7.50 b	A 5.46 b	A 6.18 b	A 6.64 b	A 5.92 b

[1] FB = fall burn; FYM =Farmyard manure; NB, NB45 and NB90 = No burn with N applied at 0 kg ha^{-1}, 45 kg ha^{-1}, and 90 kg ha^{-1}, respectively; PV = pea vine; SB, SB45 and SB90 = spring burn with N applied at 0 kg ha^{-1}, 45 kg ha^{-1}, and 90 kg ha^{-1}, respectively.

[2] Upper case letters show comparison of the 10 treatments within each year and depth, and [3] lower-case letters show comparison of the four depths within each of the nine treatments. Means sharing the same letter are not significantly different at 5% level of significance.

Supplementary Table S5. Mean concentration of soil total nitrogen (g N kg^{-1}) obtained from the 108 combinations of treatment, year and depth after 84 years in the crop residue long-term experiment (CR-LTE), Pendleton, OR.

Depth					Treatment				
(cm)	FB[1]	FYM	NB	NB45	NB90	PV	SB	SB45	SB90
**1995**									
0-10	C[2] 0.72 a[3]	A 1.37 a	BC 0.88 ab	B 0.92 ab	B 0.95 ab	B 1.01 a	BC 0.86 a	B 0.93 a	B 0.95 a
10-20	C 0.76 a	A 1.31 a	BC 0.92 a	BC 0.95 a	B 0.97 a	B 1.03 a	BC 0.89 a	BC 0.95 a	B 0.98 a
20-30	A 0.85 a	A 0.91 b	A 0.92 a	A 0.92 ab	A 0.89 ab	A 0.93 ab	A 0.88 a	A 0.89 ab	A 0.88 ab
30-60	A 0.69 a	A 0.73 b	A 0.71 b	A 0.73 b	A 0.76 b	A 0.77 b	A 0.76 a	A 0.72 b	A 0.74 b
**2005**									
0-10	C 0.75 a	A 1.18 a	BC 0.92 a	BC 0.92 a	BC 0.92 a	B 0.97 a	BC 0.83 a	BC 0.85 ab	BC 0.92 a
10-20	B 0.77 a	A 1.09 a	B 0.85 ab	B 0.87 ab	B 0.88 a	AB 0.94 a	B 0.87 a	B 0.88 a	B 0.84 ab
20-30	A 0.75 a	A 0.85 b	A 0.78 ab	A 0.74 ab	A 0.83 a	A 0.82 ab	A 0.80 ab	A 0.77 ab	A 0.79 ab
30-60	A 0.70 a	A 0.72 b	A 0.66 b	A 0.68 b	A 0.67 b	A 0.73 b	A 0.67 b	A 0.68 b	A 0.68 b
**2015**									
0-10	C 0.76 a	A 1.30 a	BC 0.89 a	BC 0.86 ab	BC 0.88 ab	B 1.00 a	BC 0.81 a	BC 0.89 a	BC 0.88 a
10-20	C 0.69 a	A 1.20 a	BC 0.85 a	B 0.92 a	B 0.91 ab	B 0.97 a	BC 0.78 a	B 0.88 a	BC 0.86 a
20-30	C 0.74 a	A 1.25 a	C 0.83 a	BC 0.91 a	BC 0.94 a	B 1.04 a	C 0.79 a	BC 0.88 a	BC 0.85 a
30-60	A 0.67 a	A 0.76 b	A 0.68 b	A 0.71 b	A 0.71 b	A 0.68 b	A 0.70 a	A 0.73 a	A 0.71 a

[1] FB = fall burn; FYM =Farmyard manure; NB, NB45 and NB90 = No burn with N applied at 0 kg ha^{-1}, 45 kg ha^{-1}, and 90 kg ha^{-1}, respectively; PV = pea vine; SB, SB45 and SB90 = spring burn with N applied at 0 kg ha^{-1}, 45 kg ha^{-1}, and 90 kg ha^{-1}, respectively.

[2] Upper case letters are comparison of the nine treatments within each year and depth, and [3] lower-case letters are comparison of the four depths within each of the nine treatments. Means sharing the same letter are not significantly different at 5% level of significance.
[4] NA = not available.

Supplementary Table S6. ANOVA table of the main and interaction effects of year and treatment on the accumulation of N, C, S, P, K, Ca, and Mg in the grain and straw of wheat in the crop residue long term experiment. Significant effects (p-value < 0.05) that require multiple means comparison are shown in bold.

Source of variation	Nitrogen	Carbon	Sulfur	Phosphorous	Potassium	Calcium	Magnesium
				Wheat Grain			
Year (Y)	0.01	0.61	0.04	0.14	0.97	0.11	0.15
Treatment (T)	<0.01	**<0.01**	<0.01	**<0.01**	**<0.01**	**<0.01**	**0.02**
Y × T	**<0.01**	0.27	**0.02**	0.27	0.52	0.52	0.48
				Wheat Straw			
Year (Y)	0.05	0.10	0.02	0.90	0.18	0.39	0.02
Treatment (T)	<0.01	**0.01**	<0.01	0.36	<0.01	0.02	<0.01
Y × T	**<0.01**	0.18	**<0.01**	**0.02**	**<0.01**	**<0.01**	**<0.01**

Supplementary Table S7. Multiple means comparisons of the concentration of N and S accumulation in the wheat grain as affected by the interaction of treatment and year.

Year	NB[1]	NB45	NB90	FB	SB	SB45	SB90	PV	FYM
					N (g kg^{-1})				
1995	B[2]12.26 d[2]	C 13.35 bcd	B 16.62 a	B 11.46 d	B 12.01 d	A 13.36 bcd	A 13.36 bcd	B 15.06 abc	A 15.34 ab
2005	B 12.38 de	B 15.67 b	AB 19.17 a	B 11.87 e	B 11.92 e	A 15.01 bc	A 15.01 bc	A 18.81 a	A 14.15 bcd
2015	A 13.32 c	A 17.70 abc	A 20.72 ab	A 13.40 c	A 13.49 c	A 16.19 bc	A 16.19 bc	A 18.50 abc	A 21.75 a
					S (g kg^{-1})				
1995	A 0.64 ab	A 0.92 ab	A 0.84 ab	A 0.95 ab	B 0.91 ab	A 0.91 ab	A 0.91 ab	A 0.81 b	B 1.09 a
2005	A 0.95 abc	A 0.81 bc	A 0.79 bc	A 0.93 abc	B 0.74 c	A 0.82 bc	A 0.82 bc	A 0.79 bc	B 1.11 a
2015	A 1.01 bc	A 1.01 bc	A 0.92 bc	A 1.04 bc	A 1.06 bc	A 0.88 c	A 0.88 c	A 0.82 c	A 1.52 a

[1] NB= No burn, SB= spring burn, FB= fall burn, FYM= farmyard manure, and PV= pea vine. 0, 45, 90 accompanied by NB, SB, and FB represents rates of N applied at 0 kg ha-1, 45 kg ha-1, and 90 kg ha-1, respectively.

Means sharing the same letter are not significantly different at 5% level of significance.

[2] Upper case letters are comparison of the 3 years within each of the 9 treatments (for year × treatment interaction) and lower-case letters are comparison of the 9 treatments within each of the 3 years (for year × treatment interaction).

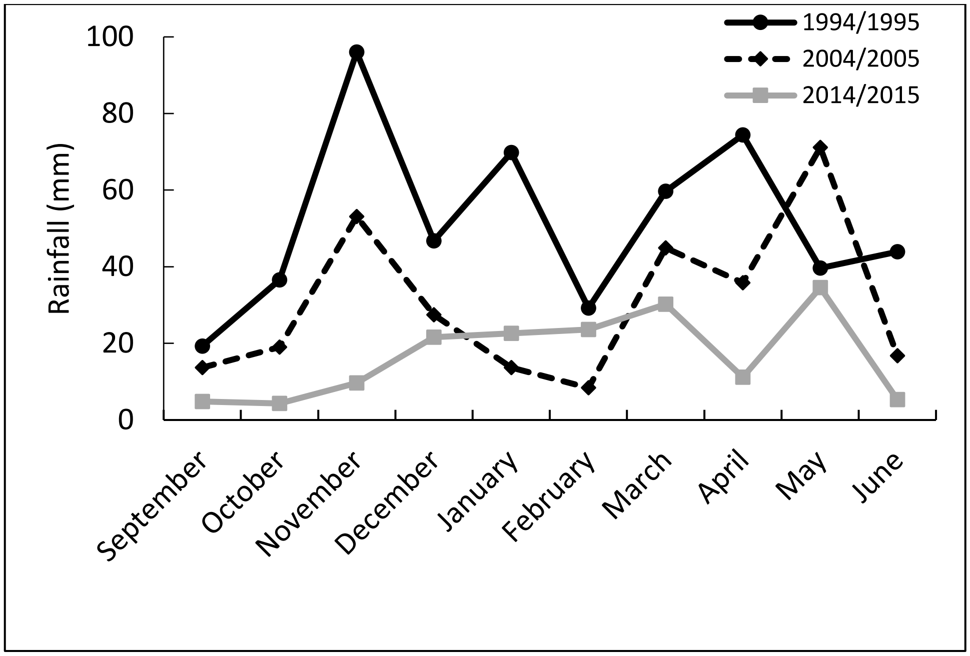

Supplementary Fig. S1. Rainfall in winter wheat growing season in Pendleton, Oregon.
